# A Case Series of Patients With Polyneuropathy Due to Copper Deficiency

**DOI:** 10.7759/cureus.37329

**Published:** 2023-04-09

**Authors:** Ulviyya Gasimova, Prerana Jayanth, Mona Kafaie

**Affiliations:** 1 Neurology, Saint Louis University School of Medicine, St. Louis, USA; 2 Biology, Saint Louis University, St.Louis, USA

**Keywords:** alcohol abuse, gastric bypass surgery, peripheral neuropathy, copper deficiency, malnutrition

## Abstract

We are presenting six cases of patients with peripheral polyneuropathy due to malnutrition in settings of prior history of gastric bypass surgery, zinc-based dentures usage, or long-standing alcohol abuse. The clinical presentation in all six patients included sensory, motor, or combined peripheral polyneuropathy and gait instability due to imbalance. All patients included in this case series were found to have low copper levels. Electromyography (EMG) with nerve conduction study (NCS) showed predominantly axonal and length dependent sensory or sensory-motor polyneuropathies. Patients were treated with copper supplements with reportable improvement in their presenting symptoms.

## Introduction

Many known risk factors may lead to malnutrition, such as alcohol abuse, eating disorders, gastrointestinal (GI) system disorders or surgeries, chronic liver failure, prolonged total parenteral nutrition (TPN) dependence, etc. These risk factors may affect all aspects of the nervous system, and the peripheral nervous system is no exception. Peripheral neuropathies related to malnutrition may have various timelines to develop and may be sensory, motor, or combined with either axonal or demyelinating nature with length-dependent presentation. Nutritional deficiencies related to the abovementioned causes include different vitamin or element deficiencies. It is worth mentioning that copper deficiency is rare to come across [1-4]. Copper deficiency is mainly seen in patients with increased zinc intake or post gastric bypass surgeries leading to malabsorption of copper from the GI tract as per newer studies [5,6]. Copper deficiency may lead to peripheral polyneuropathy, myelopathy, or a combination of both, affecting the quality of daily life activities in the adult population. With this case series, our primary goal is to increase awareness of malnutrition and its complications. Timely diagnosis and treatment are essential.

## Materials and methods

This is a retrospective study that includes six patients who presented with signs and symptoms of peripheral polyneuropathy. The study was conducted at Saint Louis University Hospital, St. Louis, Missouri, United States. Patients were recruited between the years 2020 and 2022. Data were obtained from electronic medical records. Neurological exams of patients were consistent with sensory or combined length-dependent polyneuropathies. They all were found to have low copper levels as a result of risk factors leading to impaired absorption.

## Results

The first patient was a 46-year-old lady with a past medical history of left adrenal adenoma on computer tomography (CT), gastric bypass surgery, and zinc-based denture cream use who was noted to also have a five-year history of syncopal episodes alongside numbness and tingling that originated in the soles of her feet and eventually spread up to her knees. Additional symptoms included muscle cramps and bilateral lower extremity (BLE) weakness. Physical exam was notable for symmetrically decreased light touch (LT), pinprick (PP), and temperature sensation in BLE in length-dependent nature with intact strength and vibration/position sense. The copper level was found to be undetectable. She was started on treatment with weekly intravenous copper infusions. During the follow-up visit, she reported a decrease in the frequency of her syncopal events and improvement in her BLE muscle weakness and sensory symptoms.

The second patient was a 62-year-old man with a past medical history of zinc-based denture cream use and more than 10 years history of alcohol abuse who presented with less than a year of weakness of BLE and unsteady gait. On physical exam, he was noted to have symmetrically length-dependent diminished LT, PP, temperature, and vibration sense in BLE with intact strength. Due to low copper levels and risk factors for malnutrition, a 30-day course of oral copper gluconate was prescribed. Unfortunately, we could not confirm the improvement in his symptoms as he lost to follow-up. 

The third patient was a 61-year-old woman with a past medical history of gastric bypass surgery who presented to the clinic with stiffness in BLE, bilateral weak hand grip, dysphagia, hypotonic voice, and falls due to imbalance. Physical exam was remarkable for mildly decreased strength 4/5 and spasticity in bilateral upper and lower limbs with intact sensory exam findings. She was found to have low copper and vitamin B12 levels, most likely due to malabsorption in settings of prior history of gastric bypass surgery. As per exam findings, this patient had a combination of myeloneuropathy in settings of vitamin and element deficiencies. She was prescribed oral copper and vitamin B12 supplementations. The patient is currently scheduled for a follow-up. 

The fourth patient was a 42-year-old woman with a history of gastric bypass surgery, Crohn’s disease with small bowel resection, and use of zinc-containing denture cream who presented with a worsening three-year history of numbness and tingling that originated in the bottom of the left foot that eventually spread to her bilateral feet. She subsequently developed bilateral foot drop and worsening global muscle weakness, which resulted in wheelchair use due to her inability to ambulate. Physical exam revealed a decreased LT, PP, and temperature sensation in BLE and 3/5 strength in distal lower extremities with preserved strength in bilateral upper extremities (BUE) and proximal BLE. Vibration was noted to be 2/8 bilaterally at knees. An extended workup for her presenting symptoms was completed which showed copper of less than 10. Eventually, with intravenous copper infusions, gabapentin, pregabalin, and regular physical therapy visits, the patient’s global muscle weakness improved. On the follow-up visit, the patient could ambulate with a walker.

The fifth patient was a 33-year-old woman with a medical history of gastric bypass surgery and alcohol use who was admitted to the hospital with two weeks history of generalized weakness that initially manifested as falls and eventually progressed to being unable to ambulate unassisted. On physical exam, she was noted to have 3/5 strength in upper limbs proximally and distally and 4/5 strength in lower limbs proximally and distally length dependent with decreased LT and temperature sensation in BLE up to ankles. Dysmetria was noted on the finger-nose-finger test. Cerebrospinal fluid (CSF) was obtained due to concern for Guillain-Barre syndrome (GBS), but inflammatory as well as infectious workup came back normal with normal glucose level at 53, protein level at 18, xanthochromia negative, lymphocytes 86%, monocytes 6%, macrophages 3%, red blood cells 0, and white blood cells 2. The copper level was low at 61. Based on the results, essential electrolytes and vitamins were repleted, and copper supplementation was ordered. However, the patient never picked up the copper supplementations and was lost to follow-up. 

The sixth patient was a 24-year-old woman with a one-year history of eating disorder and presumed diagnosis of myasthenia gravis (MG) with acetylcholine receptor (AchR) blocking antibody. The patient was diagnosed with MG due to proximal weakness in her upper and lower limbs. She was hospitalized multiple times over the last year with similar symptoms and was previously discharged from a different hospital a week prior to the current episode. She was treated with intravenous immunoglobulin (IVIG) during these admissions with no improvement. Initial symptoms at our hospital included the inability to bear weight and difficulty breathing. During admission, the patient endorsed symptoms of burning and tingling in BLE that were present since the original MG diagnosis. Physical exam was notable for 2/5 strength in upper and lower limbs proximally and distally. Further workup revealed low serum copper levels at 34. Electromyography (EMG) and nerve conduction study (NCS) were remarkable for severe sensorimotor length-dependent polyneuropathy with axonal features without findings consistent with MG. Oral copper supplementation was started with significant improvement in global strength. Laboratory workups for common causes of peripheral neuropathy including common metabolic, autoimmune, and infectious causes were unrevealing as well as thyroid function tests.

The risk factors and laboratory findings for all the cases are shown in Tables 1, 2. 

**Table 1:** The risk factors for malnutrition leading to copper deficiency

Patient	Age	Risk Factors	Presenting Symptoms
1	46	Gastric bypass surgery, zinc-based denture cream	Small fiber neuropathy
2	62	Use of zinc-based denture adhesive, alcohol abuse	Large fiber length-dependent polyneuropathy
3	61	Gastric bypass surgery	Falls due to imbalance
4	39	Gastric bypass surgery, zinc-containing denture adhesive	Small fiber neuropathy
5	33	Gastric bypass surgery, alcohol abuse	Acute onset of generalized weakness and falls
6	24	Eating disorder	Burning and tingling sensation with profound weakness

**Table 2:** Laboratory values Reference range for copper: 72-166 mcg/dL; Reference range for vitamin B12: 213-816 pg/ml; Reference range for hemoglobin A1C: <=5.6% RPR: rapid plasma reagin

Patient	Copper level (mcg/dL)	Vitamin B12 level (pg/ml)	Hemoglobin A1C level (%)	HIV and RPR tests	Treatment
1	<5	366	4.9	Non-reactive	Intravenous copper infusions
2	64	465	4.7	Non-reactive	30-day course of copper Gluconate
3	74	941	6.6	Non-reactive	Oral copper supplementation
4	<10	676	5.1	Non-reactive	Intravenous copper infusion
5	61	872	4.0	Non-reactive	Oral copper supplementation
6	34	853	5.2	Non-reactive	Oral copper supplementation

All six patients were found to have results consistent with predominantly axonal sensory or sensorimotor length-dependent polyneuropathy on EMG and NCS. Table 3 gives the sensory nerve amplitude potentials (SNAP) and conduction velocities for radial and sural nerves. Table 4 gives the reference values for the same.

**Table 3:** EMG and NCS study findings SNAP: sensory nerve amplitude potential; EMG: electromyography; NCS: nerve conduction study; NR: no read; L: left; R: right

Patient	Age	SNAP Amplitudes (µV)		Conduction Velocities (m/s)	
		Radial	Sural	Radial	Sural
1	46	31.9 (L)	5.1 (L), 6.7 (R)	62 (L)	40 (L), 46 (R)
2	62	20.2 (R)	6.4 (L), 6.4 (R)	47 (R)	32 (L), 36 (R)
3	61	24.0 (R)	NR (L), NR (R)	59 (R)	NR (L), NR (R)
4	39	27.7 (L)	10.6 (L), 7.7 (R)	61 (L)	54 (L), 59 (R)
5	33	6.2 (L) 8.9 (R)	3.4 (L), 5.2 (R)	53 (L) 65 (R)	58 (L), 51 (R)
6	24	NR (L)	NR (L), NR (R)	NR (L)	NR (L), NR (R)

**Table 4:** Reference values for conduction study SNAP: sensory nerve action potential; CV: conduction velocity

Variables	Reference values
Radial SNAP (µV)	>=15
Sural SNAP (µV)	>=5
Radial CV (m/s)	>=53
Sural CV (m/s)	>=45

## Discussion

Copper deficiency may result from various causes such as malnutrition, malabsorption, or impaired copper transport. The exact mechanism by which copper deficiency leads to neurological consequences is not clearly known; however, copper is a cofactor in various oxidative enzymes. Copper-containing metalloenzyme is a component of the electron transport chain that is part of adenosine triphosphate (ATP) production and participates in oxidative phosphorylation, which is an important step in myelin production [7].

There are several case reports discussing the neurological outcomes of copper deficiency. One of the case reports published by Amin and co-authors highlighted the case of peripheral neuropathy due to malnutrition/malabsorption in settings of prior gastric bypass surgery. The diagnosis was initially thought to be a chronic inflammatory demyelinating disease, and the patient underwent extensive workup. As the authors mention, the leading diagnosis was delayed due to delayed workup for malnutrition as a leading cause of the patient’s presenting symptoms. After the diagnosis was made and the patient was started on copper supplementation, the symptoms were noted to improve [8].

Griffith et al. presented two cases of peripheral neuropathy in patients with a remote history of gastric bypass surgery who were found to have a copper deficiency. Patients were treated with copper supplementations, and authors reported a quite rapid improvement in hematologic abnormalities, whilst neurological symptoms resolved only mildly. The authors mention that the neurological outcomes of treatment can be variable and depend on the route and degree of copper supplementations [9].

In the present case series, the main goal is to highlight the importance of timely workup for nutritional causes of peripheral neuropathy in patients complaining of sensory, motor, or combined neuropathies or myeloneuropathies with a subacute or chronic timeline of onset. Especially patients with risk factors for malnutrition or malabsorption should be in a high-risk category. It is important to note that patients recruited to our study developed neuropathic symptoms within three years after gastric bypass surgery or zinc-based denture use. 

The limitation of this case series is the number of patients included in this study. Larger studies or case series on this topic are needed to understand the discussed etiology and provide better care for patients. Thinking of how many patients with peripheral polyneuropathy each neurologist sees throughout their career brings up the question of what else we could do for this population to ease their struggle. Timely diagnosis and appropriate treatment not only improve the symptoms but are also likely to prevent neurological complications.

## Conclusions

Through this case series, we have shown the various clinical manifestations of copper deficiency such as peripheral neuropathy (paresthesias, decreased sensations, and imbalance) with or without weakness (deficiencies in gait and muscle cramps) as well as one case of combined myeloneuropathy. Peripheral neuropathies related to malnutrition may be related to a single risk factor or a combination of multiple factors as presented in our case series. Malnutrition is a very well-known risk factor leading to various vitamin deficiencies including copper deficiency, which may cause peripheral polyneuropathy, myelopathy, or a combination of both. Our case series is not the first one to highlight these clinical sequelae; however, with the recent trend of gastric bypass surgeries and the tendency to overuse alcohol, it is not enough to increase awareness of malnutrition.

Through these cases, we once more want to highlight the importance of checking plasma copper levels in patients that present with neuropathic symptoms, especially in the setting of zinc-based denture use, history of gastric bypass surgery, alcohol abuse, or any risk factors that may lead to malnutrition. Excessive alcohol use-related vitamin and element deficiencies are more frequent scenarios nowadays as the tendency to overuse alcohol is becoming common not only in the older generation but also among youth. An appropriate approach in a timely manner may significantly improve symptoms that affect the quality of daily life and may prevent further neurological deterioration. Keeping copper deficiency in mind while taking care of patients with polyneuropathy may help to determine the underlying cause earlier and provide an opportunity to offer a timely cost-effective medical approach as well. 
